# The Utility of Fat Grafting to Manage Burn Scars: A Systematic Review

**DOI:** 10.1093/jbcr/iraf146

**Published:** 2025-07-18

**Authors:** Omar El Sewify, Xi Ming Zhu, Cory Tremblay, Eolie Delisle, Shahriar Shahrokhi

**Affiliations:** Faculty of Medicine, Laval University, Quebec City, QC G1V 0A6, Canada; Division of Plastic Surgery, McMaster University, Hamilton, ON L8S 4L8, Canada; Faculty of Medicine, Northern Ontario School of Medicine, Sudbury, ON P3E 2C6, Canada; Division of Otolaryngology, University of Montreal, Montreal, QC H3C 3J7, Canada; Division of Surgery, McMaster University, Hamilton, ON L8S 4L8, Canada

**Keywords:** fat grafting, burn scars, adipose derived stem cells, scar remodeling

## Abstract

Secondary management of thermal injuries remains a challenging and relevant topic for plastic and nonplastic burn surgeons alike. Burn scars are associated with both functional limitations and aesthetic challenges for patients. While various treatment modalities exist for the management of these scars, no gold standard has been established. Fat grafting has been used in various reconstructive contexts, and studies have demonstrated improvement in skin texture and contour following infiltration. This systematic review aims to examine the available evidence on outcomes following fat grafting for the management of burn scars. A search of Medline, EMBASE, and Cochrane Library databases was conducted from their inception until November 2024. Published articles examining outcomes of fat grafting for thermal injury scars were identified, screened, and data were extracted following Preferred Reporting Items for Systematic Reviews and Meta-Analyses guidelines. A total of 10 740 articles were screened, yielding 14 eligible studies for data extraction, accounting for 885 patients. All studies reported improvement of scars postoperatively. Nine out of 14 studies used a subjective clinical assessment, 1 study did not report pretreatment measurements, and the other 8 studies all found improved outcomes based on clinician assessment. One study reported Vancouver Scar Scale (VSS) scores and another reported modified VSS scores. Three studies utilized POSAS and the mean difference was an improvement of 7.28 (MCID <1). This review suggests that autologous fat grafting and adipose-derived stem cells show promising results for improving scar quality, function, and patient satisfaction following burn injury. Further studies, particularly prospective in nature, with standardized outcome measurements are needed to substantiate subjective clinical improvement. The authors recommend utilizing POSAS or VSS for future studies investigating burn scar treatments.

## INTRODUCTION

Burns are a major global health concern, ranking among the leading causes of death and disability worldwide. While burn injuries have been declining in high-income countries, they remain highly prevalent in low- and middle-income regions, where approximately 90% of burns occur.^1^^,^^2^ The WHO estimates around 11 million burn injuries of varying severity occur globally each year, with 180 000 resulting in fatalities.^3^ Burn management presents significant challenges, both in the acute phase and during long term recovery, particularly with regard to secondary burn scars.^4^^,^^5^ Burn scars have a profound affect on patients’ self-esteem, mental health, negatively impact their social interactions, and can have a considerable impact on their overall quality of life, making it a common challenge in outpatient clinics.^6–8^

Beyond the visible and psychological effects, burn scars often present persistent symptoms, including neuropathic pain, pruritus, impaired mobility, and the possibility of developing Marjolin ulcers.^9–12^ Severe burns can result in significant damage to subcutaneous fat and fascia with resultant adherence between skin grafts and underlying tissue. In addition, contractures can significantly impair mobility and function.^6^ Advancements in burn care have improved survival rates, thereby directing focus toward minimizing the long-term functional and esthetic consequences of burn scars to enhance patients’ quality of life.^7^

Burn care has significantly evolved, with increasing emphasis on treatments aimed at improving the long-term quality of life for burn survivors by addressing the challenges posed by scars.^6^^,^^13^ Secondary management of burn injuries encompasses a wide range of interventions, from conservative therapies to more invasive surgical options. Conservative treatments, such as scar massage, silicone gel sheeting, CO_2_ laser therapy, and intra-lesional steroid injections, have demonstrated varying degrees of success. However, their effectiveness is often limited, particularly for minor lesions or when used in isolation. These approaches tend to yield better outcomes when integrated into a multimodal strategy tailored to the patient’s unique needs.^14^

Autologous skin grafting remains the cornerstone of burn treatment.^8^ This method offers a range of techniques that can be tailored to the unique characteristics of each burn injury, patient needs, and clinical circumstances, ensuring optimal coverage and functional restoration.^15^ Furthermore, advancements in wound care products such as dressings, topical antiseptics and antimicrobials, skin substitutes, and systemic antibiotics have enhanced the overall management of burn injuries but can often be a time-consuming and slow process.^4^ While skin grafts are highly effective, they introduce additional donor-site morbidity, which leads to visible scarring, secondary contractures, and long-term complications such as pain and pruritus.^4^ Local flaps are particularly useful for the release of burn contractures, offering a valuable option for improving both function and appearance in patients with extensive scarring.^8^

Autologous fat grafting has emerged as an innovative therapeutic approach, highlighting the potential of adipose tissue and its components in promoting tissue regeneration, remodeling the extracellular matrix (ECM), and exerting antifibrotic effects.^7^^,^^8^^,^^13^ This technique involves the injection of adipose tissue into burn scars and has exhibited potential in treating scars caused by radiation therapy, thermal injuries, chronic wounds, and vascular ulcers.^4^^,^^16^ In recent years, innovative fat transplantation techniques, such as nano fat and adipose-derived stem cell (ADSC) matrix gels, have emerged, highlighting the expanding therapeutic applications of adipose tissue and its diverse regenerative potential.^17^ Its regenerative effects are largely attributed to ADSCs, the principal cellular component utilized in fat grafting.^18^ ADSCs secrete proangiogenic factors, including vascular endothelial growth factor and fibroblast growth factor, which enhance blood flow and oxygen delivery to ischemic scar tissue.^19^ They also remodel the ECM by releasing matrix metalloproteinases, reducing fibrotic collagen, and promoting deposition of healthier collagen. Additionally, ADSCs exert anti-inflammatory and immunomodulatory effects through cytokines like interleukin-10 (IL-10) and transforming growth factor-beta (TGF-β), mitigating chronic inflammation and fibrosis. These processes collectively contribute to improved scar elasticity, reduced pain, and softening of hypertrophic scars. Beyond restoring volume, ADSCs promote nerve regeneration by releasing neurotrophic factors, potentially alleviating chronic pain and restoring sensation in burn scars.^20^^,^^21^ The combination of these effects highlights the role of fat grafting as a valuable option in the secondary management of burn scars given its biocompatibility, minimally invasive nature, and ability to be easily harvested in large quantities.^16^^,^^17^^,^^22–24^

Our study aims to provide a comprehensive review of the literature on fat grafting in the secondary management of burn scars, underscoring its benefits for scar remodeling, including improvements in esthetic outcomes, pain reduction, and functional recovery.

## METHODS

This study was conducted according to the methodology described in the *Cochrane Handbook for Systematic Reviews of Interventions* and is reported according to the Preferred Reporting Items for Systematic Reviews and Meta-Analyses (PRISMA) statement.^25^ The protocol for this systematic review was registered on Open Science Framework (osf.io/rdbna).

### Identification of studies

A search was conducted between inception and November 17th, 2024, of the Medline, Embase, and Cochrane Libraries. Articles were screened by 2 authors (OE and CT), and if disagreements occurred that could not be consensually resolved, they were subsequently discussed with a senior author (XMZ).

### Eligibility criteria

Included studies reported outcomes of fat grafting for patients with burn scars. No restrictions were placed on specific outcome measures of interest. Studies on fat grafting for other injuries were excluded. Studies that looked at outcomes of varied scar etiology may be included if sufficient granularity was present in the dataset looking specifically at burn scar outcomes. No restrictions were placed on social or demographic features of the patient; however, pediatric studies (patients aged ≤17) were excluded. Similarly, no restrictions were placed on the fat processing technique. Case reports, editorials, reviews, expert opinions, basic science articles, and non-English studies were excluded.

### Data extraction

Data were extracted by 2 independent reviewers (OE and CT) using piloted screening forms. The dataset was checked over by XMZ and curated for clinical relevance. Information extracted includes author and date of publication, location of burn, fat processing technique, subjective clinical outcome assessment, and standardized burn scar assessment tools.

### Statistical analysis

Results are presented descriptively, as no pooling of data was performed given heterogeneity of outcome reporting as well as missing critical data (eg, standard deviations). Agreement between independent reviewers was assessed using the Cohen *k* statistic. Based on the guidelines of Landis and Koch,^26^ a k statistic of 0 to 0.2 represents slight agreement; 0.21 to 0.4 fair agreement; 0.41 to 0.6 moderate agreement; 0.61 to 0.8 substantial agreement, and ≥0.81 is considered almost perfect agreement. The *k* statistic was calculated using StataCorp 2023 (Stata Statistical Software: Release 18).

## RESULTS

### Study characteristics

**Figure 1:**
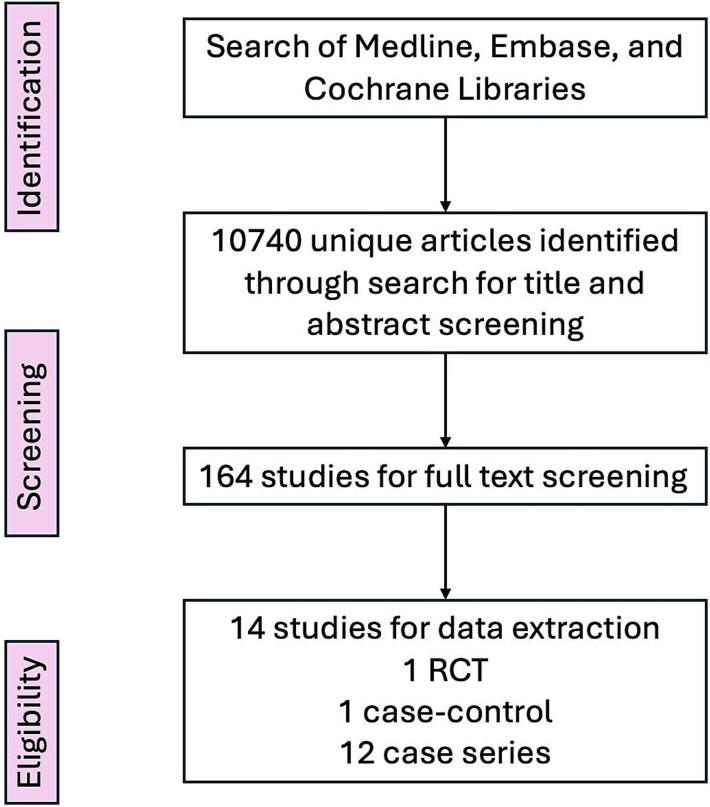
PRISMA Flow Diagram

A total of 10 740 unique articles were identified and screened. Following title and abstract screening, 164 full texts remained, of which 14 were deemed eligible for data extraction (Figure 1). The studies were composed of 1 RCT,^5^ 1 case–control,^27^ and 12 case series.^6–8^^,^^16^^,^^24^^,^^28–34^ Agreement on inclusion between reviewers was almost perfect for title and abstract (*k*, 0.887; SE, 0.020) and was almost perfect for full text screening (*k*, 0.922; SE, 0.055).

### Risk of bias assessment

Risk of bias assessment for the RCT study can be found (Table SA) in the Appendix. Overall risk of bias for this study raised some concerns, particularly with regard to deviation from the intended intervention.^5^

Risk of bias for non-RCT studies is listed in (Table SB) of the Appendix. For this study, a score of ≤8 was considered poor quality, a score of 9-14 was considered moderate, and ≥15 was considered to be good quality. Seven studies were considered moderate quality,^6^^,^^7^^,^^24^^,^^29–32^ while 6 studies were considered good quality.^8^^,^^16^^,^^27^^,^^28^^,^^33^^,^^34^ Notably, many studies lacked reporting of outcomes using standardized assessments. In addition, studies that did report using standardized scar assessment tools did not provide critical data necessary for pooled analysis, nor enough granularity to allow for its derivation.

### Subjective clinical outcomes

**Figure 2:**
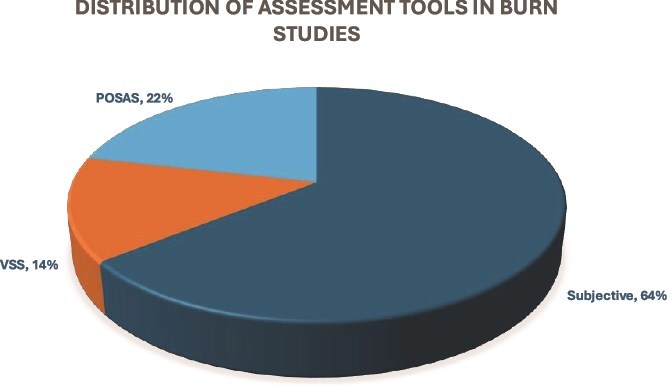
Distribution of Assessment Tools in Burn Studies

Nine out of 14 studies utilized a nonstandardized clinical assessment tool or evaluated using clinical qualitative reporting^5^^,^^16^^,^^27^^,^^28^^,^^30–34^ (Figure 2). One study did not report pretreatment baseline scores for their clinical assessment.^28^ The remaining 8 out of 9 studies all reported improvement posttreatment.

### Vancouver Scar Scale and POSAS

**Figure 3:**
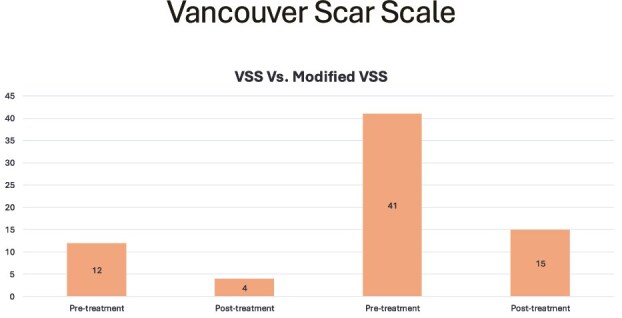
Vancouver Scar Scale

Gargano et al. reported outcomes using the Vancouver Scar Scale (VSS).^24^ The mean pretreatment score was 12, with a mean posttreatment score of 4. Bruno et al. reported outcomes using the Modified VSS and found improvement following fat grafting treatment, with a mean of 41 before treatment and 15 after treatment at latest follow-up^8^ (Figure 3).

**Figure 4:**
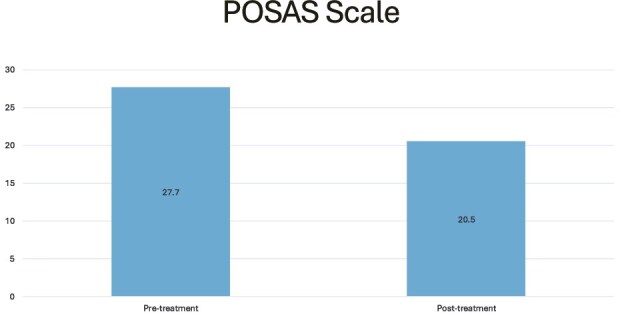
POSAS Scale

Three studies reported outcomes using the POSAS scale.^6^^,^^7^^,^^29^ These studies encompass a total of 100 patients. The pooled pretreatment mean score was 27.7, with a mean of 20.5 posttreatment (Figure 4). The lack of standard deviations and granularity in data reporting precluded the ability to perform a meta-analysis.

## DISCUSSION

Autologous fat grafting and adipose-derived stem cells have been commonly used in surgical treatments for regeneration and rejuvenation purposes. Our systematic review synthesized data from 14 studies, comprising 1 RCT,^5^ 1 case–control study,^27^ and 12 case series,^6–8^^,^^16^^,^^24^^,^^28–34^ reporting outcomes in a total of 885 patients. These studies examining the use of fat grafting or ADSCs for burn scars have shown promising outcomes, highlighting the potential of autologous fat grafting in burn scar management.

Autologous fat grafting is widely recognized in human studies for enhancing scar quality after burn injuries.^4^^,^^16^ This improvement is attributed to increased vascularity, new collagen deposition and reorganization, as well as enhanced mimic features such as color, texture, thickness, and overall patient satisfaction.^35^ A recent systematic review investigating autologous fat grafting for burn scars and their meta-analysis of 42 studies with 3033 patients revealed that roughly 90% of patients and surgeons combined were satisfied with the clinical outcomes following a mean follow-up period of 2 years.^36^ However, these reported patient and surgeon evaluation scores specific to the respective scar or burn sequelae were quantified using subjective satisfaction scores, which are subject to biases. While the previous systematic review reported on 42 studies and conducted a meta-analysis evaluating autologous fat grafting for burn scars, our review applied more rigorous inclusion criteria, focusing specifically on human subjects, isolated burn scar populations, and studies with sufficient methodological quality. Several studies included in the prior review were excluded from our analysis^37–39^ due to mixed scar etiologies, use of convenience sampling from large patient groups, or inadequate reporting of outcome measures. Although many of the studies we included used similar subjective outcome measures, there remained significant variability in patient populations, treatment protocols, and follow-up periods, which limited the feasibility of conducting a pooled analysis. Thus, this review aimed to provide a more focused synthesis of the available evidence specific to burn scar treatment.

Internationally accepted scar quality assessment tools such as POSAS and VSS enable simple and objective outcome evaluation. Studies employing the VSS^8^^,^^24^ and POSAS^6^^,^^7^^,^^29^ in our review demonstrated statistically significant improvements in scar appearance. The findings from Bruno et al. and Gargano et al. noted a reduction in the Modified VSS score from 41 to 15 posttreatment and a decrease in mean VSS score from 12 to 4, respectively.^8^^,^^24^ The findings from the previous studies indicate that fat grafting can significantly enhance patient outcomes (Appendix S1, Figures S5–S8), emphasizing its potential effectiveness in improving scar quality. Moreover, 3 studies assessed outcomes using the POSAS scale.^7^^,^^11^^,^^12^ The pooled results indicated a significant reduction in mean POSAS scores (27.7 pretreatment to 20.5 posttreatment), suggesting an overall improvement in patient-reported scar quality. This trend indicates potential clinical benefits, as reduced POSAS scores are associated with better scar texture, pigmentation, and pliability of scars. These case series align with a comprehensive systematic review and meta-analysis, demonstrating substantial overall improvements in patient assessment scores across all POSAS subcategories, except for itching.^36^ Pooled data from the POSAS observer module indicate significant improvements in scar quality and pliability.^36^ However, the mean improvements in POSAS scores exceed the minimal clinically important difference (MCID), which determines whether a change in the POSAS score is clinically meaningful.^37^ This study determined that MCID values had better discriminatory ability, indicating that patients perceive even minor POSAS score changes as clinically significant for burn scar quality.^40^ The lack of data granularity prevented pooling of data for analysis, thereby precluding the ability to confidently state whether the mean improvement is applicable. These findings can aid in evaluating treatment efficacy and guiding sample-size calculations for future research.

Fat grafting was also associated with improvements in functional outcomes, particularly in regions where scar contractures limited mobility, such as the hands or face. Abouzaid et al. showed improvement in texture, pigmentation, scar hypertrophy, and contracture when comparing the use of autologous fat grafting to conventional burn treatments.^5^ Byrne et al. described improved hand function postfat grafting, particularly when applied in combination with surgical release of scar contractures.^7^ Other authors have supported these claims, noting that using adipose-derived stromal vascular fraction reduces fibrosis and improves skin quality and functionality in late sequelae of burn scars in hands.^41^^,^^42^ These functional improvements highlight fat grafting’s role beyond esthetic benefits, potentially enhancing the quality of life and daily function of burn survivors.

Nonsurgical treatments such as scar massage, silicone gel sheets, CO_2_ laser therapy, and intralesional steroids have been used with ambiguous success. These options often provide limited benefit in isolation, and their therapeutic impact is typically enhanced when used as part of a comprehensive, individualized treatment plan.^14^ A key limitation of the current literature is the lack of comparative studies evaluating autologous fat grafting against nonsurgical scar modulation therapies, as previously mentioned. Most included studies in this review were case series without control groups, limiting our ability to determine whether fat grafting offers superior outcomes. While improvements in VSS and POSAS scores were commonly reported, these outcomes were not directly compared to those achieved with less invasive and more economical interventions. Given the higher resource burden and procedural risks associated with fat grafting, such as the need for general anesthesia, fat embolism, hematoma, and fat necrosis, it is currently not well established in the literature if these alternative therapies are of greater benefit to overall patient outcomes. Future studies should prioritize direct comparisons between surgical and nonsurgical interventions, utilizing control groups and incorporating cost–benefit analyses to better define their clinical value.

### Strengths and limitations

A key strength of this review lies in its novel exploration of the topic to formally synthesize the current literature. The amalgamation of the available data was accomplished using a rigorous methodology by screening a very large number of studies to ensure that the analysis likely captured all available evidence on the subject. This level of diligence strengthens the reliability of the findings and enhances the review’s credibility as a comprehensive resource for advancing knowledge in this field.

Our study is not without limitations. Many studies lacked critical data for a more granular analysis, such as standard deviations or follow-up details necessary to assess long-term outcomes. This gap was a notable limitation in the studies that used standardized scales like VSS^8^^,^^24^ and POSAS,^6^^,^^7^^,^^29^ as only pooled mean scores were provided, as the absence of detailed statistical data prevented a robust meta-analysis. Moreover, the absence of pretreatment baseline scores in some studies^16^^,^^27^^,^^28^ further limits the ability to accurately quantify the changes attributable to fat grafting. Additionally, 9 out of 14 studies^5^^,^^16^^,^^27^^,^^28^^,^^30–34^ used nonstandardized qualitative assessments, which introduces potential biases and limits the comparability of findings. Despite these limitations, there was a consistent trend toward patient-reported improvement, suggesting a broad potential benefit of fat grafting in burn scar management. The discrepancy in measurement methods and the subjective nature of some assessments mean that these results should be interpreted with caution. The risk of bias assessment indicated concerns primarily with the RCT, specifically regarding deviations from the intended interventions. This highlights the importance of rigorous adherence to protocols in future studies. For the non-RCT studies, the quality varied, with 7 studies^6^^,^^7^^,^^24^^,^^29–32^ deemed moderate quality and the remaining 6 studies were considered good quality.^8^^,^^16^^,^^27^^,^^28^^,^^33^^,^^34^ This variability emphasizes the need for improved reporting standards, particularly regarding outcome measures. Many studies failed to utilize standardized assessment tools, which limits the comparability of results and could obscure the true effectiveness of fat grafting techniques. While nearly all documented uses of this therapy for human burn scars report positive outcomes, much of the existing literature is based on case reports or case series that lack rigorous statistical analysis or sufficient power. The absence of standardized clinical assessment tools calls into question the reliability and generalizability of these results, and future studies should utilize a large, prospective, randomized, controlled trial investigating its use in patients with burn scars. This review is inherently limited by the quality and scope of the included studies, many of which lacked comparator groups and relied on small sample sizes using subjective scales such as VSS and POSAS. For example, Gargano et al. reported a reduction in mean VSS scores from 12 to 4 following treatment, while Bruno et al. observed an improvement in Modified VSS scores from 41 to 15 after fat grafting.^8^^,^^24^ These findings suggest potential benefit, but remain limited by subjective evaluation tools and small sample sizes. Furthermore, while we report on 105 patients utilizing scar assessments using VSS and Modified VSS tools^8^^,^^24^ and roughly 100 patients using POSAS^7^^,^^13^^,^^29^ in our cumulative cohort, the reliance of nonstandardized outcome measures limits the generalizability of our findings. Future research should incorporate comprehensive data reporting to confirm these observations, supporting broader clinical applications of treatments evaluated with these standardized scales.

## CONCLUSION

This systematic review suggests that autologous fat grafting and ADSCs show promising results for improving scar quality, function, and patient satisfaction following burn injury. While clinical outcomes indicate enhancements in vascularity, collagen deposition, and functional mobility, limitations in study design, standardized assessment, and reporting standards call for more rigorous research. Future large-scale, randomized controlled trials with standardized outcome measures, control groups, and direct comparisons between surgical and nonsurgical interventions are essential to validate these findings and establish autologous fat grafting as a reliable treatment option for burn scars.

## Supplementary Material

Appendix_1_(figures5_6_7_8)_iraf146

Appendix_1_iraf146

Appendix_RB_iraf146

## Notes

Author contributions: Omar El Sewify (Conceptualization, Data curation, Formal analysis, Funding acquisition, Investigation, Methodology, Project administration, Resources, Software, Supervision, Validation, Visualization, Writing—original draft, Writing—review & editing [lead]), Xi Ming Zhu (Methodology, Project administration, Software, Supervision, Writing—original draft, Writing—review & editing [equal]), Cory Tremblay (Data curation, Formal analysis, Investigation, Methodology, Writing—original draft, Writing—review & editing [equal]), Éolie Delisle (Data curation, Formal analysis, Methodology, Writing—original draft, Writing—review & editing [equal]), and Shahriar Shahrokhi (Writing—original draft, Writing—review & editing [equal])

Funding: None declared.
