# Supplementary material for: The Utility of Fat Grafting to Manage Burn Scars: A Systematic Review
Source: J Burn Care Res. 2025 Jul 18;46(6):1269–75. doi: 10.1093/jbcr/iraf146 (PMC12596688; doi:10.1093/jbcr/iraf146)
Supplement: Appendix_1_iraf146 [file appendix_1_iraf146.docx]

Appendix 1

This appendix consists of images from the findings from Bruno et al. (2013) and Gargano et al. (2018) utilizing autologous fat grafting to treat burn sequelae, where they noted reductions in the Modified VSS score from 41 to 15 post-treatment and a decrease in mean VSS score from 12 to 4, respectively [8, 24].


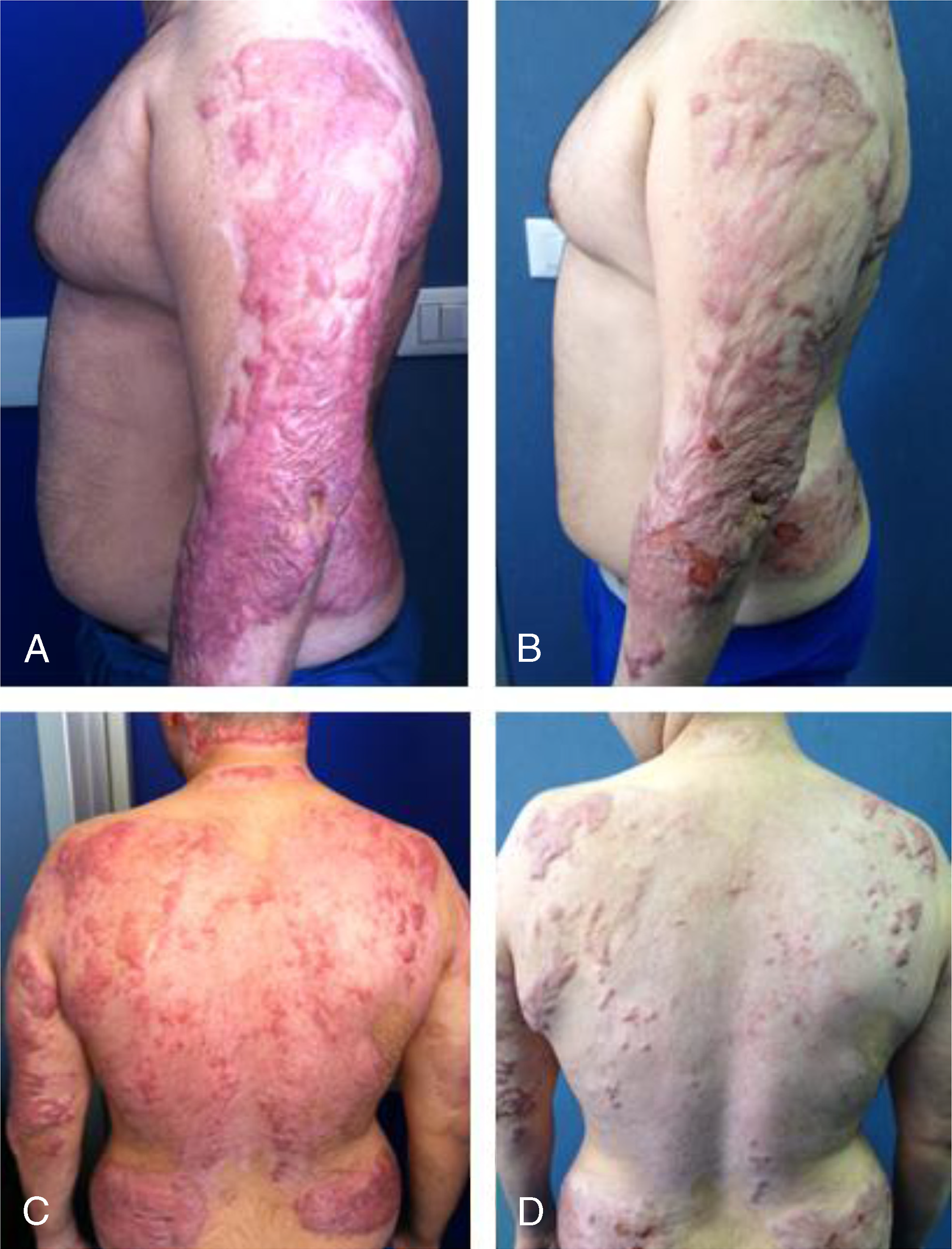


**Figure 5**. This figure, adapted from Bruno et al. (2013) [8], demonstrates burn scars prior to treatment (A, C) and following six months of fat grafting (B, D), highlighting significant functional and aesthetic improvement in the hypertrophic scars.


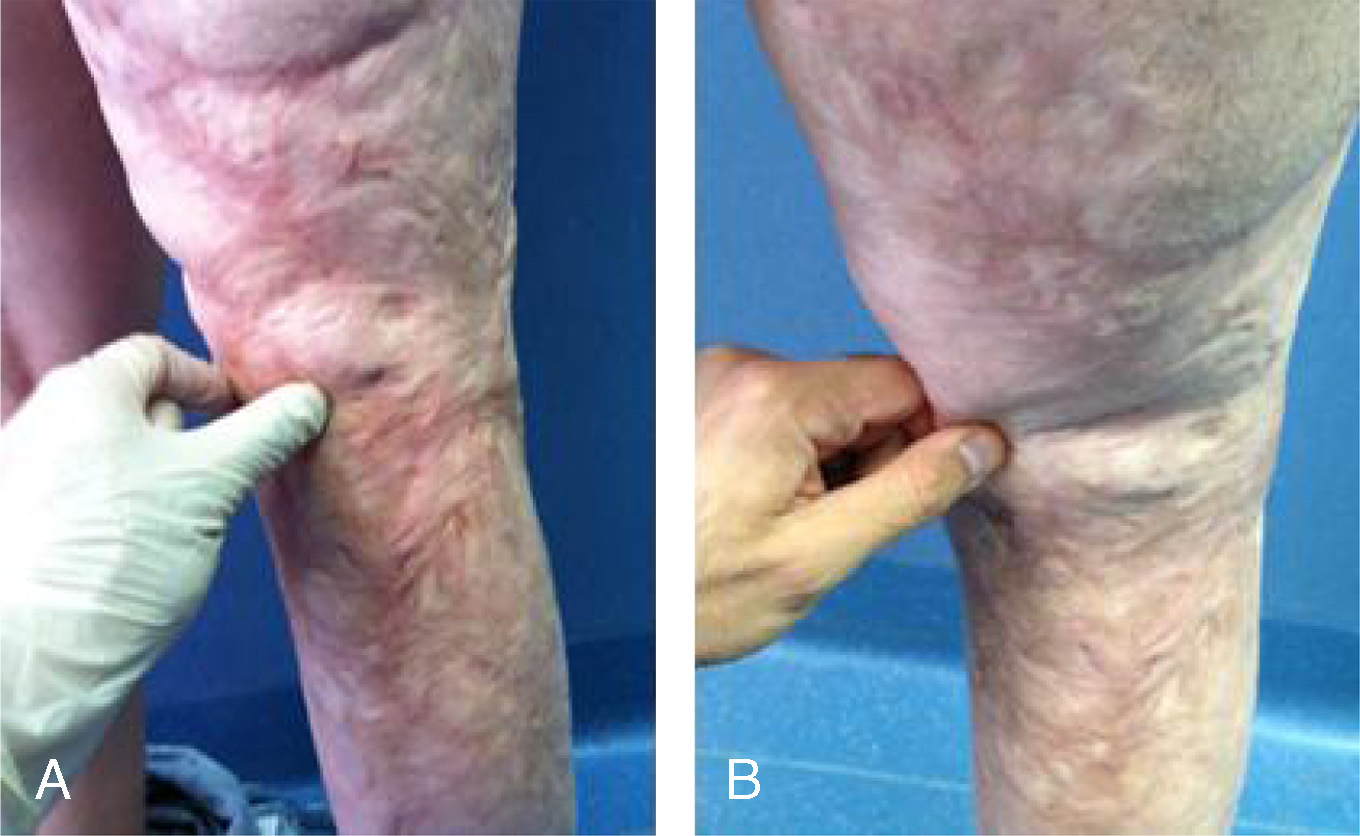


**Figure 6**. This figure, adapted from Bruno et al. (2013) [8], depicts the scar prior to treatment (A) and following treatment (B), highlighting improved function, aesthetics, and tissue pliability.


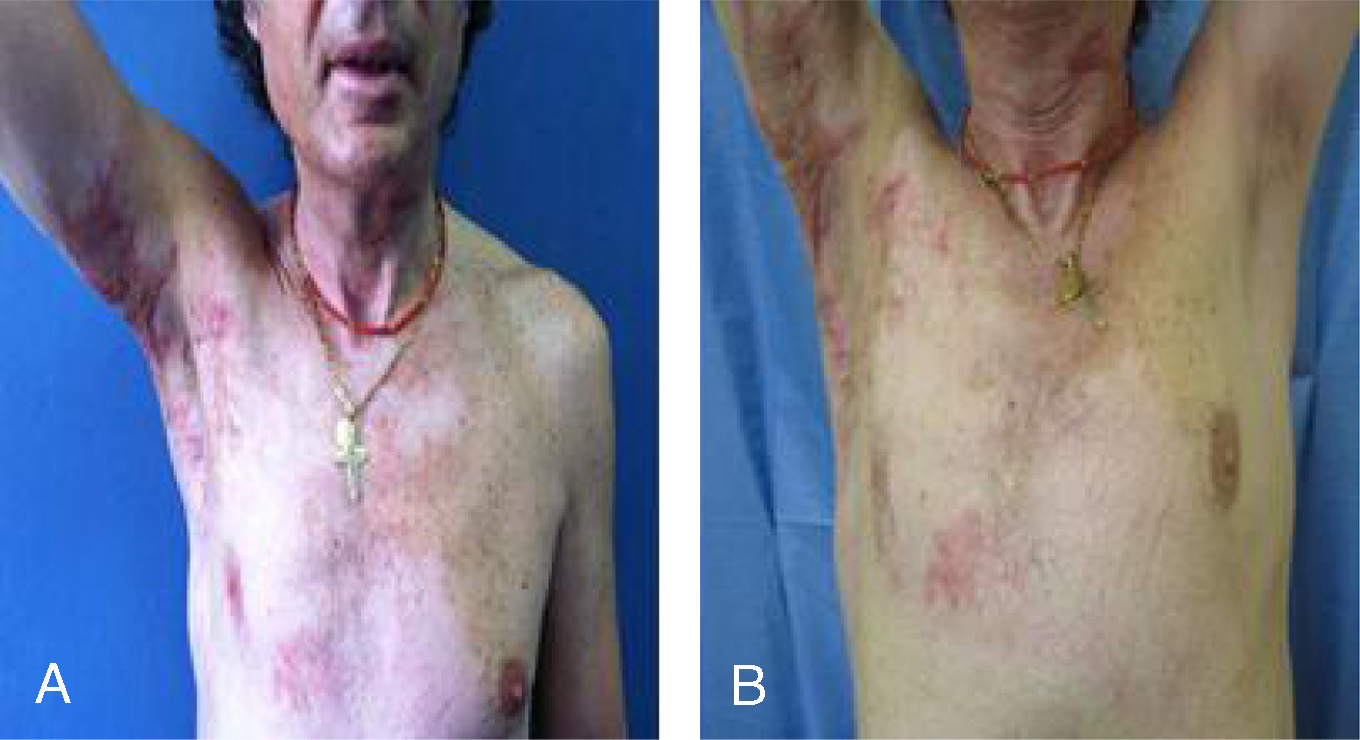


**Figure 7**. This figure, adapted from Bruno et al. (2013) [8], shows the patient pre-treatment (A) and six months post-treatment following fat grafting and rigottomies (B), demonstrating restored limb extension. No Z-plasties were performed.


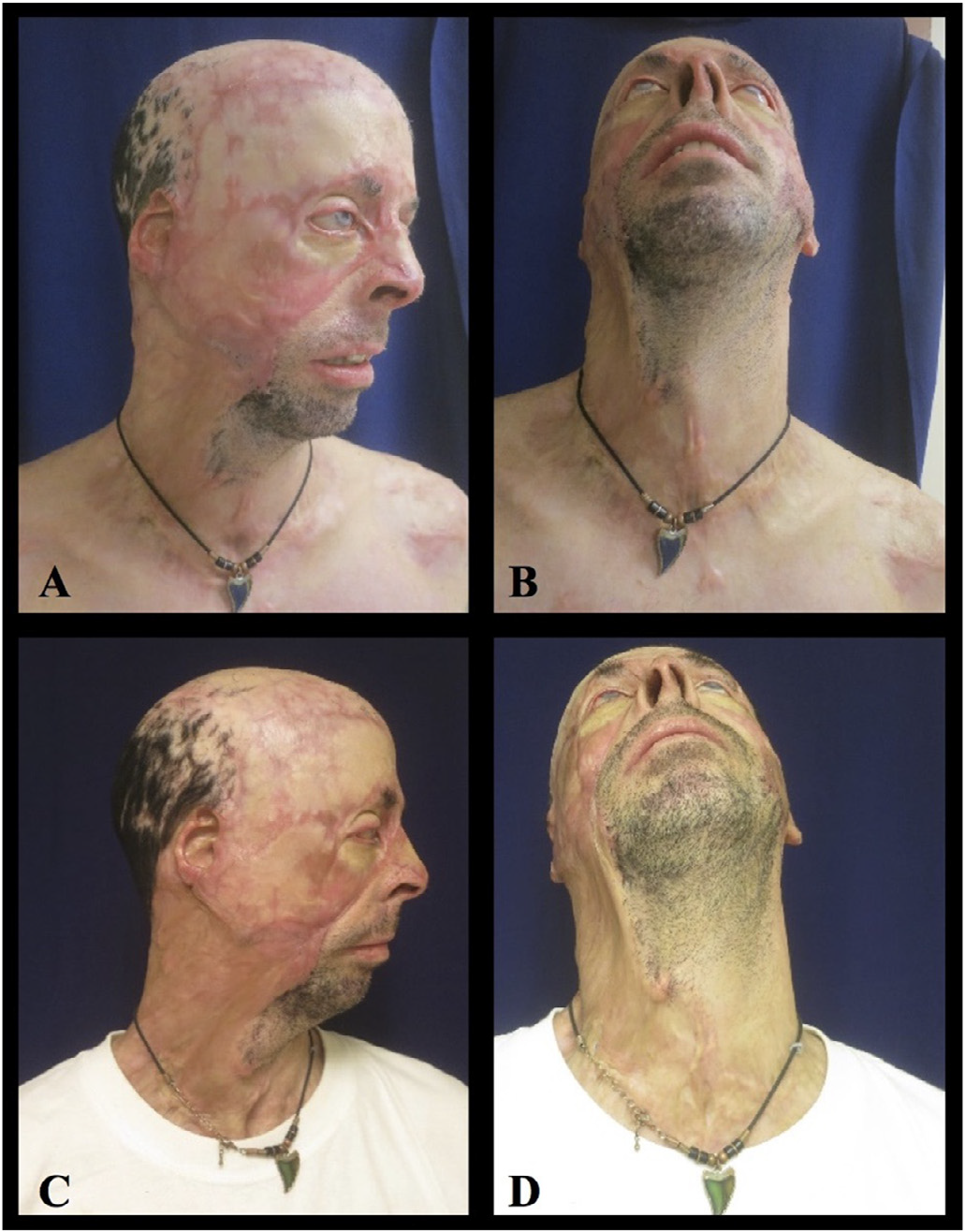


**Figure 8**. This figure, adapted from Gargano et al. (2018) [24], illustrates range of motion pre-operatively (A, B) and at 12 months post-operatively (C, D) following autologous fat grafting
