# Supplementary material for: The Utility of Fat Grafting to Manage Burn Scars: A Systematic Review
Source: J Burn Care Res. 2025 Jul 18;46(6):1269–75. doi: 10.1093/jbcr/iraf146 (PMC12596688; doi:10.1093/jbcr/iraf146)
Supplement: Appendix_RB_iraf146 [file appendix_rb_iraf146.docx]

APPENDIX

Table A.

RoB2 assessment of randomized controlled studies

| Study | Risk of bias from randomization | Risk of bias due to deviations from intended interventions | Missing outcome data | Risk of bias in measurement of the outcome | Risk of bias in selection of the reported result | Overall risk of bias |
| --- | --- | --- | --- | --- | --- | --- |
| Abouzaid et al 2022 | High | High | Low | Low | Low | Some concerns |

Table B.

MINORs evaluation of non-RCT studies

| Study | Clearly stated aim | Inclusion of consecutive patients | Prospective collection of data | Endpoints appropriate to the aim of the study | Unbiased assessment of study endpoint | Follow-up period appropriate to aim of study | Loss to follow-up <5% | Prospective calculation of study size | Adequate control group | Contemporary groups | Baseline equivalence of groups | Adequate statistical analysis | Total |
| --- | --- | --- | --- | --- | --- | --- | --- | --- | --- | --- | --- | --- | --- |
| Bruno et al. 2013 | 2 | 2 | 1 | 2 | 0 | 2 | 0 | 0 | 2 | 2 | 2 | 2 | 17 |
| Byrne et al. 2016 | 2 | 1 | 1 | 2 | 0 | 2 | 0 | 0 | 0 | 2 | 0 | 2 | 12 |
| Gargano et al. 2018 | 2 | 2 | 1 | 2 | 0 | 2 | 0 | 0 | 0 | 2 | 0 | 2 | 13 |
| Gentile et al. 2014 | 2 | 2 | 1 | 2 | 0 | 2 | 0 | 0 | 2 | 2 | 1 | 1 | 15 |
| Gentile et al. 2017 | 2 | 2 | 1 | 2 | 0 | 2 | 0 | 0 | 2 | 2 | 1 | 2 | 16 |
| Gentile et al. 2023 | 2 | 1 | 1 | 2 | 0 | 2 | 0 | 0 | 2 | 2 | 1 | 2 | 15 |
| Jan et al. 2018 | 2 | 2 | 1 | 2 | 0 | 2 | 0 | 0 | 0 | 2 | 0 | 2 | 13 |
| Jaspers et al. 2017 | 2 | 2 | 1 | 2 | 0 | 2 | 0 | 0 | 0 | 2 | 0 | 2 | 13 |
| La Padula et al. 2018 | 2 | 2 | 1 | 2 | 0 | 2 | 0 | 0 | 0 | 2 | 0 | 1 | 12 |
| Piccolo et al. 2015 | 2 | 1 | 1 | 2 | 0 | 2 | 0 | 0 | 0 | 2 | 0 | 0 | 10 |
| Piccolo et al. 2020 | 2 | 1 | 1 | 2 | 0 | 2 | 0 | 0 | 0 | 2 | 0 | 0 | 10 |
| Xu et al. 2018 | 2 | 1 | 1 | 2 | 0 | 2 | 0 | 0 | 2 | 2 | 1 | 2 | 15 |
| Zhou et al. 2022 | 2 | 2 | 1 | 2 | 0 | 2 | 0 | 0 | 2 | 2 | 1 | 2 | 16 |
